# Quantification of arbuscular mycorrhizal fungi root colonization in wheat, tomato, and leek using absolute qPCR

**DOI:** 10.1007/s00572-023-01122-8

**Published:** 2023-08-30

**Authors:** Andrea Corona Ramírez, Sarah Symanczik, Tabea Gallusser, Natacha Bodenhausen

**Affiliations:** Department of Soil Sciences, Research Institute of Organic Agriculture FiBL, Frick, Switzerland

**Keywords:** Arbuscular mycorrhizal fungi, Quantitative PCR (qPCR), Microscopy, Crops, Absolute quantification

## Abstract

Arbuscular mycorrhizal fungi (AMF) form symbioses with most terrestrial plants and are known to have a positive effect on plant growth and health. Different methodologies have been developed to assess the AMF-plant symbiosis. The most applied method, which involves staining of roots and microscopic observation of the AMF structures, is tedious and time-consuming and the results are highly dependent on the observer. Using quantitative polymerase chain reaction (qPCR) to quantify AMF root colonization represents a reliable, high-throughput technique that allows the assessment of numerous samples. Quantification with qPCR can be performed through two methods: relative quantification and absolute quantification. In relative quantification, the target gene is normalized with a reference gene. On the other hand, absolute quantification involves the use of a standard curve, for which template DNA is serially diluted. In a previous paper, we validated the primer pair AMG1F and AM1 for a relative quantification approach to assess AMF root colonization in *Petunia.* Here, we tested the same primers with an absolute quantification approach and compared the results with the traditional microscopy method. We evaluated the qPCR method with three different crops, namely, wheat (cv. Colmetta and Wiwa), tomato, and leek. We observed a strong correlation between microscopy and qPCR for Colmetta (*r* = 0.90, *p* < 0.001), Wiwa (*r* = 0.94, *p* < 0.001), and tomato (*r* = 0.93, *p* < 0.001), but no correlation for leek (*r* = 0.27, *p* = 0.268). This highlights the importance of testing the primer pair for each specific crop.

**Supplementary Information:**

The online version contains supplementary material available at 10.1007/s00572-023-01122-8.

## Introduction

Arbuscular mycorrhizal fungi (AMF) are beneficial soil fungi which form symbiotic relationships with most terrestrial plants. AMF are characterized by their tree-shaped structures, known as arbuscules, formed after penetrating the cell wall of the host plant root (Smith and Read 2010; Heijden et al. 2015). The arbuscule is the primary location for nutrient exchange where the plant provides carbohydrates and lipids to the AMF in exchange for essential macro- and micronutrients such as phosphorus (P), nitrogen (N), iron, coper, and zinc (Walder and Van Der Heijden 2015; Keymer and Gutjahr 2018). AMF can expand the plant’s nutrient access area by colonizing large volumes of soil with their hyphal network. Understanding the abundance, growth, and activity of AMF in soil, as well as their response to agricultural practices (Boddington and Dodd 2000; Alguacil et al. 2014), soil parameters (Kahiluoto et al. 2001), and plant genetics (Parniske 2008), among other factors, is crucial because of their significant role in promoting plant growth.

Quantifying the rate of plant root colonization is a widely used indicator to assess the AMF-plant symbiosis (Smith and Read 2010). Two main methods are used to quantify root colonization by AMF, phospholipid fatty acid analysis (PLFA) (Olsson et al. 1995) and microscopy (McGonigle et al. 1990). PLFA requires the extraction from plant roots of fatty acids which subsequently are analyzed by gas chromatography coupled with mass spectrometry. The phospholipid 16:1ω5 is used as a biomarker for AMF (Olsson et al. 1998). However, this biomarker also is present in small quantities in Gram-negative bacteria, and its concentration can differ among AMF species (Graham et al. 1995). Additionally, only a few samples can be analyzed in parallel, and it requires expensive equipment that not every laboratory can afford. On the other hand, microscopy-based methods require the staining of AMF structures inside the roots. For this, the roots are cleared in boiling KOH and stained with common ink (Vierheilig et al. 2005) or trypan blue (Phillips and Hayman 1970), a carcinogenic dye. Once the AMF structures are stained, root colonization can be quantified using one of several methods. One of them, the “gridline intersect” method, uses low magnification (from 7× to 50×) to observe roots placed in a petri dish with grid lines. The presence or absence of AMF is recorded for approximately 100 intersections of the gridlines with the roots (Giovannetti and Mosse 1980). While a large amount of the root system can be examined, this method has the limitation that small structures like arbuscules cannot be observed at low magnification. A second method includes a measure of colonization intensity, with five classes for root colonization and three classes for arbuscules/vesicles (Trouvelot et al. 1986). A third method is called the “magnified intersection” method (McGonigle et al. 1990), in which small root fragments are carefully mounted on microscope slides to evaluate 100 root intersections at high magnification (from 20× to 250×). At this magnification, different mycorrhizal structures can be identified, but the careful preparation of the slides is laborious. In general, microscopy methods are time-consuming, and the microscopic evaluation is subjective as it strongly depends on the experience of the observer. Thus, for the correct identification of AMF structures, proper training by experienced researchers is needed.

Molecular techniques, particularly those relying on the polymerase chain reaction (PCR), have gained popularity in the last two decades for studying AMF colonization. These techniques include the quantification of abundance using quantitative PCR (qPCR) and the characterization of community composition through amplicon sequencing. Some qPCR methods focus on the quantification of a few AMF species using specific primers (Alkan et al. 2004; Thonar et al. 2012; Symanczik et al. 2015; Heller and Carrara 2022), while others use general AMF primers to target the full diversity of AMF (Gollotte et al. 2004; Hewins et al. 2015). Primers usually target nuclear markers (Thonar et al. 2012), but some primers have also been designed to target mitochondrial markers (Badri et al. 2016; Voříšková et al. 2017).

Compared to microscopy, qPCR methods offer several advantages. First, qPCR is a high-throughput technique and is time-efficient. Second, although molecular techniques require specific training, the results are independent of the observer. Finally, the qPCR product can be sequenced to not only obtain AMF abundance but also to study their community composition (Bodenhausen et al. 2021). This advantage becomes evident when investigating the success of mixed inocula in order to assess the competitiveness of individual AMF strains within the mixture. By contrast, assessing AMF diversity though microscopy is impossible, as there are not enough morphological characteristics of colonization to discriminate among species (Abbott 1982; Merryweather and Fitter 1998). Thus, using qPCR for the quantification and community assessment of root colonization reduces the number of analyses needed to assess the AMF-plant symbiosis.

There are two primary methods for normalizing qPCR data: relative quantification and absolute quantification. With relative quantification, target gene results are normalized in relation to a reference gene (Pfaffl 2001), and the outcome is expressed as a ratio (such as AMF gene copies per plant gene copies). On the other hand, with absolute quantification, the copy number of the target gene is calculated by comparing the PCR signal to a standard curve (Brankatschk et al. 2012). For AMF studies, the results often are presented in gene copies per gram of roots or per ng of DNA (Schmittgen and Livak 2008). Consequently, absolute quantification relies on the careful weighing of the roots used for DNA extraction or the quantification of the DNA extracts. Spiking, which occasionally is used in absolute qPCR, involves the addition of an internal standard (i.e., linearized plasmid of a known sequence and concentration) to the sample before DNA extraction (Green and Field 2012). The internal standard then is amplified with a different set of primers than those of the target gene allowing for the quantification of inhibition during DNA extraction and amplification (Thonar et al. 2012). Both relative quantification and spiking require a second PCR reaction with a different primer pair (targeting either the plant gene or the internal standard) and therefore are more costly in terms of material and time. For this reason, absolute quantification is preferred. For this study, absolute qPCR with and without spiking were compared to assess their advantages for the evaluation of AMF root colonization in different crops.

In our previous study, we used the primer pair AMG1F (Hewins et al. 2015) and AM1 (Helgason et al. 1998) to quantify the colonization of plant roots by AMF in two *Petunia* cultivars. We compared the relative qPCR with the traditional microscopical quantification and found strong correlation between the two methods (Bodenhausen et al. 2021). Building on this previous study, the goal of the present work was to validate the primer pair AMF1 and AMG1F for the quantification of AMF root colonization in different agricultural crops. However, here we use absolute qPCR instead of relative qPCR. Absolute qPCR was selected for two main reasons: first, absolute quantification is more convenient when working with different species as it does not require a second PCR targeting a plant gene, which needs to be designed and validated for each species; second, fewer reactions and reagents are needed with absolute quantification. The spiking method developed with primers targeting specific fungus species was applied here to assess its applicability in AMF quantification with primers AMF1 and AMG1F, which target all AMF. The importance of the AMF symbiosis in promoting plant growth underscores the need for a high-throughput methodology to quantify its abundance in key crops like wheat, because understanding this symbiosis will be valuable to improve sustainable food production. For this reason, three different crops were selected for the test: winter wheat (*Triticum aestivum*), tomato (*Solanum lycopersicum*), and leek (*Allium porrum*). To evaluate the efficacy of molecular quantification, an AMF colonization gradient was created using the well-known negative effect of phosphorus (P) on AMF plant colonization (Abbott 1982; Nagy et al. 2009). The plant roots then were analyzed with both the traditional microscopical technique and qPCR.

## Materials and methods

### Pot experiment

To evaluate the qPCR method, three different crops were selected: winter wheat (*Triticum aestivum*) with two cultivars (cv. Wiwa, cultivar for organic agriculture, and cv. Colmetta, cultivar for conventional agriculture), tomato (*Solanum lycopersicum* cv. Marmande), and leek (*Allium porrum* cv. Carentan). Soil was collected from a meadow in Dompierre (46°51′02.6″, N 6°58′41.4″E) Fribourg, Switzerland. The location was selected due to the low P content of the soil (Olsen P: 10.3 mg/kg). The alkaline and calcareous loamy soil had a pH of 7.7; other soil properties are presented in Table S1. The plants were grown in 1 L pots, except for the leek plants which were grown in 0.5 L pots. These pots were filled with native soil and sand in a 4:1 ratio (v:v). The wheat pots (Colmetta and Wiwa) were planted with three plants per pot, the leek pots contained five plants, and the tomato pots only one plant.

A gradient of P fertilization was applied to the four crops to obtain an AMF colonization gradient. The gradient consisted of 0, 10, 25, 50, 100, 300, 450, and 600 mg of P per kg of dry substrate, applied to each crop in the form of a potassium phosphate (HK_2_PO_4_) solution. Additionally, to prevent other nutrition deficiencies, the plants received a modified no-P Hoagland (Gamborg and Wetter 1975) and Knop (9.9 mM KNO_3_, 1.02 mM MgSO_4_, 4.24 mM Ca(NO_3_)_2_) solution to a total input rate of 100 mg N/kg soil and 160 mg K/kg soil. Watering frequency increased starting from once a week to every second day depending on the demand. Demineralized water was added to saucers beneath pots until soil saturation to avoid leaching of nutrients form the pots. In contrast, fertilizer solutions all were applied to the surface using a dispenser always before watering. The experiment was performed with six replicates per fertilization level (P1–P8), per crop (Colmetta, Wiwa, tomato, and leek), resulting in 48 pots per crop.

The experiment was performed in a climate chamber (A. Schleiss AG, Magden, Switzerland) with a day/night cycle of 16/8 h with 280,000 lux, a temperature of 22°C and a relative humidity of 65% for a growth period of 84 days. The pots were randomly distributed and rotated twice a week. Each plant received 10 mL (5 mL for leek) of P fertilizer solution once a week, and the pots were watered to soil saturation with demineralized water according to crop demand.

The plants were harvested after 84 days of growth. The shoot was cut above the soil surface and weighed. After drying for 48 h at 55 °C, the dry weight was recorded. The P concentration of the shoot was analyzed following the Murphy and Riley (1958) method. The root system of each plant was carefully freed from the soil, washed, weighted, and cut in 1–2 cm segments. The roots were then divided into three equivalent subsamples, one for the AMF root staining, one for DNA extraction, and one back-up. The roots for AMF root staining were placed in a 15 mL centrifuge tube, covered with 50% ethanol, and stored at 4 °C. For DNA extraction, the root subsamples were dried at 85 °C for 2–3 h in paper bags that then were stored at room temperature in plastic boxes until further analyses. The weight of the dried subsample was recorded to calculate the root dry weight.

### Root staining for the identification of AMF

Roots were stained using the modified protocol from Vierheilig et al. (2005). Roots were rinsed with deionized water and cleared with 10% KOH for 25 min (15 min for leek) at 80 °C. After rinsing with deionized water, roots were placed in a 1% HCl solution for 1 h to neutralize the pH and subsequently were stained with an ink-vinegar solution (57 mL Black Parker Quink in 1000 mL vinegar) for 15–25 min at 80 °C. Finally, roots were rinsed with deionized water and stored in 50% glycerol. Twenty-five root fragments were mounted on a microscope slide and the presence of vesicles, arbuscules, and hyphae counted at four different points along each root piece (100 intersections in total) following the magnified intersections method (McGonigle et al. 1990) with a light Leitz Laborlux S microscope (Ernst Leitz Wetzlar GmbH, Germany) with 250× magnification. Colonization is the percent of non-negative intersections counted by microscopy.

### DNA extraction

For DNA extraction, dry roots were placed in a 2 mL reaction tube to which two 5 mm metal beads were added. After 2 h in a freezer at − 80 °C, the tubes were shaken in a TissueLyser II (Qiagen, Hilden, Germany) at 30 Hz for 3 min. DNA was extracted from 30 mg ground roots with the DNeasy® Plant Mini Kit (Qiagen, Hilden, Germany) according to the manufacturer’s recommendation. To control the efficiency of the DNA extraction and to test for the presence of qPCR inhibitors, each sample was spiked with an internal DNA standard (IS). To each sample, 10 μL of the IS (APA9 plasmid, 105 copies/μL) was added at the lysis step of the DNA extraction method. The APA9 plasmid, from here on referred as IS, was prepared according to Thonar et al. (2012). DNA concentration was quantified with a NanoDrop 2000 (Thermo Scientific, Waltham, Massachusetts, United States) and diluted 10 times in molecular grade water.

### AMF quantification with qPCR

For the quantification of AMF, we used the primer pair AMG1F (Hewins et al. 2015) and AM1 (Helgason et al. 1998) which amplify a 230 bp fragment of the AMF rSSU (ribosomal small subunit). The reaction volumes were 15 μL and contained 7.5 μL of KAPA SYBR Fast qPCR Kit Master Mix 2× Universal (Axonlab, Baden, Switzerland), 0.4 μL of each primer, and 1.5 μL of DNA template. A standard curve was prepared by diluting a plasmid which was made by cloning a PCR product amplified with the primer pairs AML1/2 (Lee et al. 2008) from spores of *Rhizoglomus irregulare*. The sequence of the standard can be found in Table S2. The qPCR assays were run in triplicate on a CFX96 Real-Time System (Bio Rad, Hercules, California), and standard dilutions were run on every plate. The qPCR program consisted of an initial denaturation step of 3 min at 95 °C, followed by 40 cycles of denaturation at 95 °C for 10 s, annealing at 62 °C for 30 s, and elongation at 72 °C for 20 s followed by a melt curve analysis (from 65 to 95 °C). Amplification efficiencies were estimated by preparing dilution series for each crop. PCR efficiency was calculated based on the slope of the dilution series linear regression and was found to be 96% for Colmetta, 85% for Wiwa, and 82% for both tomato and leek.

For the quantification of the IS (plasmid of a fragment of cassava mosaic virus DNA), the reaction volume and composition were the same except for the primer pair APA9 forward/reverse (Thonar et al. 2012). The qPCR program consisted of an initial denaturation step of 3 min at 95 °C, followed by 34 of denaturation at 95 °C for 10 s, annealing at 52 °C for 15 s and elongation at 72 °C for 20 s, followed by a melt curve analysis (from 55 to 95 °C).

The analysis of the qPCR data was conducted using the CFX Manager™ 3.1 Software (Bio Rad, Hercules, California). The qPCR values obtained from the AMF amplification were corrected using the IS amplification by first calculating the Recovery Factor (RF) for each sample as follows:

$$\mathrm{RF}=\frac{\mathrm{IS\,copy\,number\,quantified}}{\mathrm{Number\,of\,IS\,copies\,spiked}}$$

Then, the RF was used to calculate the corrected AMF copy numbers per sample:

$$\mathrm{AMF}\;\mathrm{copy}\;\mathrm{number}\;\mathrm{per}\;\mathrm{mg}\;\mathrm{of}\;\mathrm{root}=\frac{\mathrm A\mathrm M\mathrm F\,\mathrm c\mathrm o\mathrm p\mathrm y\,\mathrm n\mathrm u\mathrm m\mathrm b\mathrm e\mathrm r\,\mathrm q\mathrm u\mathrm a\mathrm n\mathrm t\mathrm i\mathrm f\mathrm i\mathrm e\mathrm d\ast\mathrm{EV}\ast\mathrm{DF}}{\mathrm R\mathrm o\mathrm o\mathrm t\,\mathrm d\mathrm r\mathrm y\,\mathrm w\mathrm e\mathrm i\mathrm g\mathrm h\mathrm t\,\left(\mathrm{mg}\right)\ast\mathrm{RF}}$$

where EV is the elution volume of the DNA (in this case 25 μL) and DF is the dilution factor (in this case 10×).

### Statistical analysis

The R statistical environment (R version 4.2.3) was used for data analysis (R Core Team 2020) with the package ggplot2 for plotting (Wickham 2016). The data were inspected to check whether they satisfied normality assumptions using residual diagnostic plots, and data transformation was used if needed (qPCR data were log-transformed and colonization data were arcsine square root transformed). One-way ANOVA followed by Tukey’s honestly significant difference (HSD) test was used to test for statistical differences between groups, and the package multcompView was used to add the significance letters to the plots. The correlation between colonization and qPCR was tested using Pearson correlations.

## Results

In this study, we designed an experiment with a gradient of AMF colonization to best compare two AMF quantification methods (qPCR and microscopy). In a pot experiment with soil poor in P, an AMF colonization gradient was established by applying a P fertilization gradient with eight levels of 0, 10, 25, 50, 100, 300, 450, and 600 mg P/kg of dry soil. The effect of the P fertilization gradient was first confirmed by comparing crops’ shoot dry weights. Figure S1 presents the well-known positive effect of P on plant growth for all four crops (Colmetta, Wiwa, tomato, and leek) reflected by an increase in shoot dry weight with increased P input (Supplemental Table S3). The increase in shoot dry weight reaches a plateau around 100 mg P/kg of dry soil for both wheat cultivars and tomato (Fig. S1A–C). In the case of leek, the plateau is reached at 300 mg P/kg (Fig. S1D). Similar trends were observed for root dry weights (Fig. S2) and shoot P uptake (Fig. S3).

**Fig. 1:**
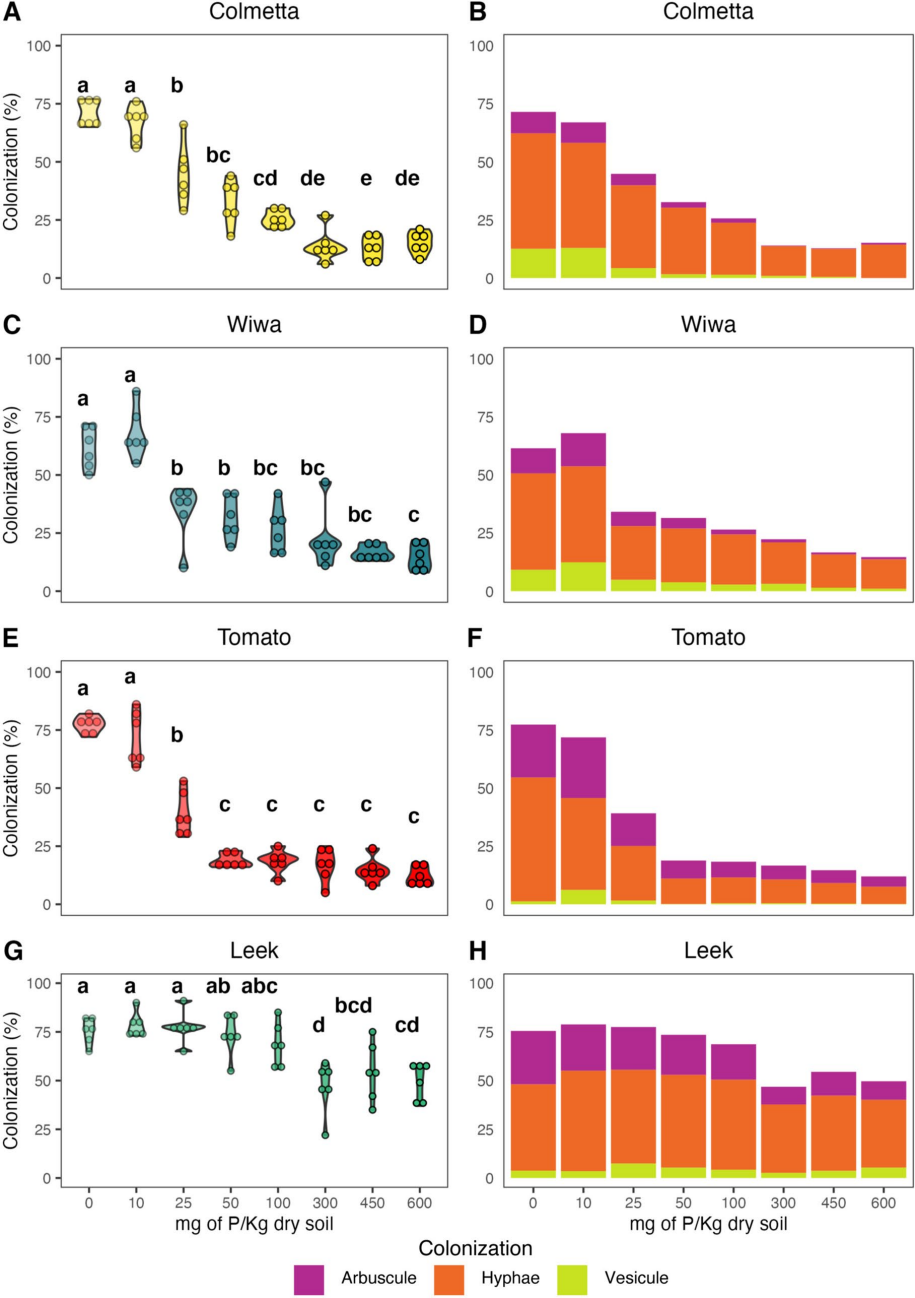
Root length colonization by arbuscular mycorrhizal fungi (AMF) estimated visually by microscopy for the winter wheat varieties Colmetta (**A**, **B**) and Wiwa (**C**, **D**), tomato (**E**, **F**), and leek (**G**, **H**). In plots **A**, **C**, **E**, and **G**, each point represents a replicate, and the color intensity increases with increasing P fertilization level. Treatments topped by the same letter do not differ significantly according to Tukey’s honestly significant difference test. Plots **B**, **D**, **F**, and **H** show the mean colonization per treatment for arbuscules (magenta), hyphae (orange), and vesicles (yellow)

Next, we used the well-established microscopy method to quantify AMF colonization. Our results showed that the root colonization was affected by P fertilization (Supplemental Table S3), as anticipated. The negative effect of P fertilization on the colonization rate can be observed in Colmetta, Wiwa, and tomato (Fig. 1 A, C, E). The highest colonization rates were observed in 0 and 10 mg P/kg of dry soil, where the mean colonization ± standard deviation was 69.3 ± 6.7% for Colmetta, 64.8 ± 10.1% for Wiwa, and 74.6 ± 8.6% for tomato. The AMF colonization ranged from 6 to 77% with SD = 23.3 in Colmetta, 8% to 86% with SD = 20.8 in Wiwa, and 5% to 86% with SD = 25.9 in tomato. By contrast, the effect of P fertilization on AMF colonization in leek presented a different response (Fig. 1G) with colonization rates ranging from 22 to 91% with SD = 15.7, thus presenting a smaller change in colonization than for the other plant varieties. Furthermore, the root colonization in leek is not affected by the level of fertilization until the P input reaches 300 mg P/kg at which the root colonization decreases from 68.7% ± 11.1 in 100 mg P/kg of dry soil to 46.8% ± 13.3 in 300 mg P/kg of dry soil.

The microscopic method not only allows the quantification of the root colonization, but also the quantification of the different AMF structures present in the plant roots. By analyzing the type of AMF structures, we observed that the proportion of each structure also changed with the P input (Table S4). Figure 1B, D, F, H shows the different AMF structures for each crop. In general, vesicles are less abundant than arbuscules or hyphae. For Colmetta, Wiwa, and tomato (Fig. 1B, D, F), there is a significant reduction of all three structures as the P concentration increases. In the case of leek, only arbuscules show a significant reduction as the P concentration increases (Fig. 1H, Table S4) while the abundance of both hyphae and vesicles did not change. A more detailed representation of Fig. 1C, D, F, H is presented in Fig. S4.

**Fig. 2:**
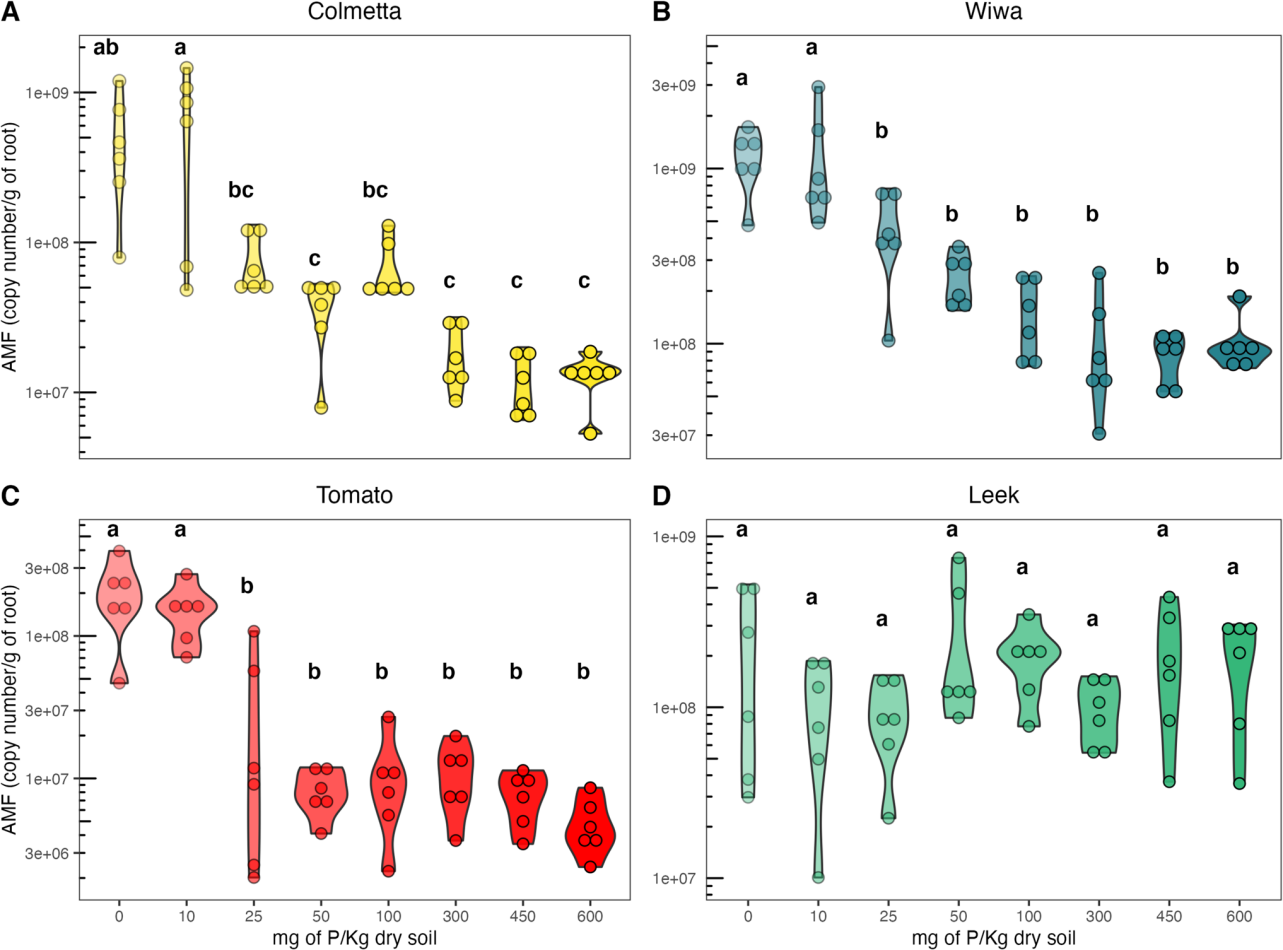
Arbuscular mycorrhizal fungal (AMF) copy numbers in roots of the winter wheat varieties Colmetta (**A**) and Wiwa (**B**), tomato (**C**), and leek (**D**) assessed by quantitative polymerase chain reaction (qPCR). Phosphorus fertilization treatments included 0, 10, 25, 50, 100, 300, 450, and 600 mg of P per kg dry soil. Each point represents a replicate, and the color intensity increases with increasing P fertilization level. Treatments topped by the same letter do not differ significantly according to Tukey’s honestly significant difference test

**Fig. 3:**
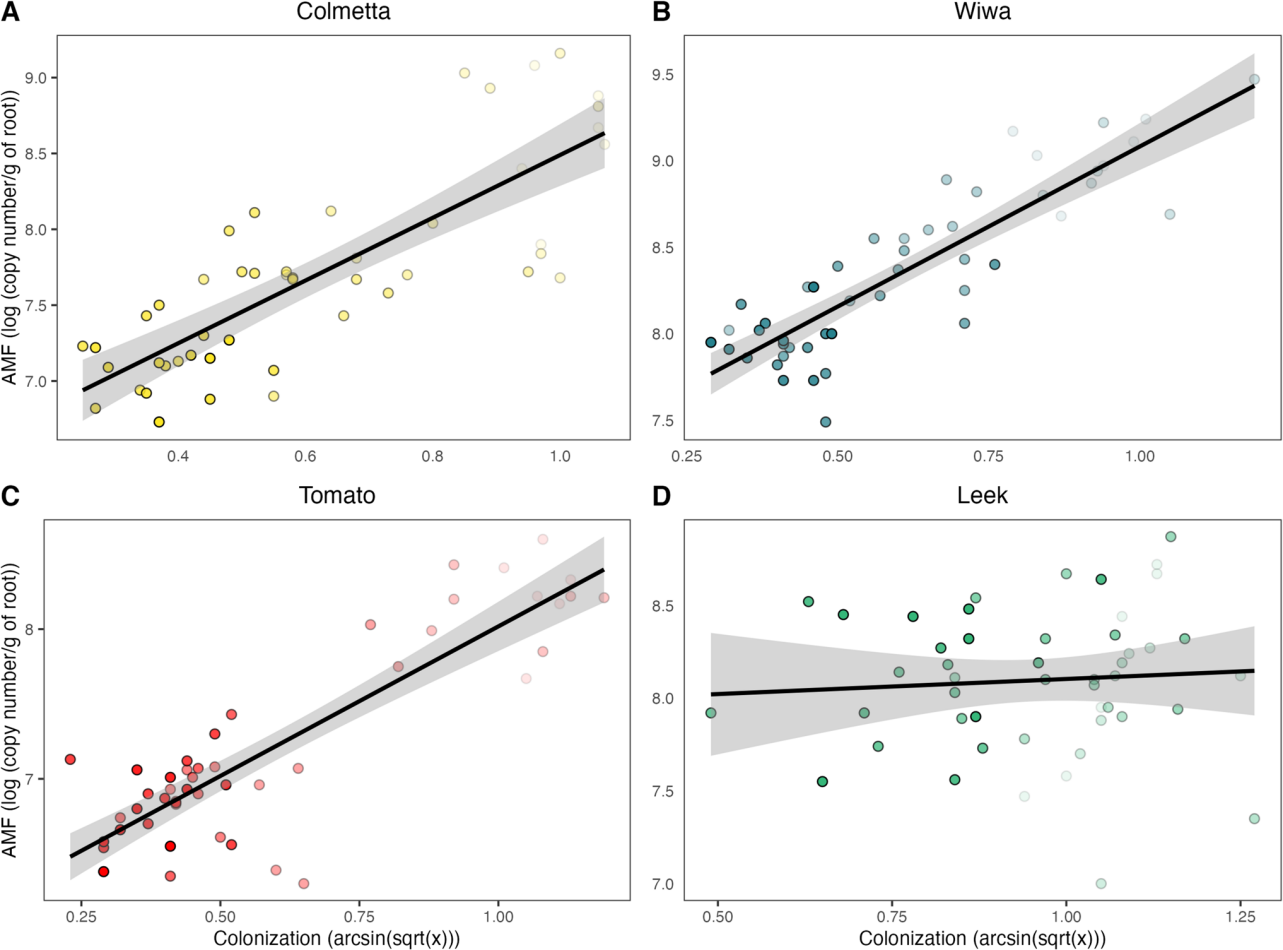
Correlation between root length colonization by arbuscular mycorrhizal fungi (AMF) determined microscopically and the abundance of AMF copy numbers quantified by quantitative polymerase chain reaction (qPCR) in the winter wheat varieties Colmetta (**A**) and Wiwa (**B**), tomato (**C**), and leek (**D**). Each point represents a replicate, and the color intensity increases with increasing phosphorus fertilization level

We used AMF primers AMG1F and AM1 to quantify the overall AMF root colonization and found it also to be affected by the P gradient (Table S3). In Colmetta, Wiwa, and tomato (Fig. 3A–C), AMF abundance significantly decreased with the addition of 25 mg P/kg and remained unchanged with further additions of P, from 50 to 600 mg P/kg of dry soil (Fig. 2). On the other hand, the AMF abundance in leek showed no significant changes across all fertilization levels (Table S3). Furthermore, the mean copy abundance of AMF was the highest in Wiwa (4.01 × 10^8^ ± 5.69 × 10^8^ copy number/g root) compared to Colmetta, tomato, or leek (1.79 × 10^8^ ± 3.38 × 10^8^, 5.41 × 10^7^ ± 8.79 × 10^7^, and 1.76 × 10^8^ ± 1.51 × 10^8^ copy number/g root, respectively).

In this study, the qPCR spiking method was used to correct for DNA extraction and qPCR amplification inhibition (Thonar et al. 2012). This method included adding an IS to each sample prior to DNA extraction and quantifying its concentration in an independent qPCR reaction. The results (Fig. S5) show that the gene copy numbers changed in comparison to the uncorrected data (Fig. 2), but the observed trends did not change. Thus, the next analysis was performed with the absolute values (without correction with the spiking method).

Finally, a linear regression model was used to compare the qPCR-based AMF quantification with the traditional microscopy root staining technique (Fig. 3). The analysis showed a significant correlation for Colmetta (*r* = 0.90, *p* < 0.001), Wiwa (*r* = 0.94, *p* < 0.001), and tomato (*r* = 0.93, *p* < 0.001), but no correlation was found for leek (*r* = 0.27, *p* = 0.268). The scatterplots of Colmetta and Wiwa followed a uniform distribution of the data points along the regression line, while for tomato, two clusters were observed for low P samples (Fig. 3, light red) and high P samples (dark red). A more detailed representation of Fig. 3 is presented in Fig. S6.

## Discussion

In this study, we tested the qPCR method to quantify AMF root colonization developed by Hewins et al. (2015) for its use in different crops including two winter wheat varieties, tomato, and leek. qPCR quantification is a high-throughput methodology that could complement or even replace traditional microscopic AMF analysis, but to be a good alternative, results obtained by qPCR need to reflect those obtained by microscopy. Previous studies have shown significant correlations between qPCR and microscopy using specific primers targeting single AMF species which were inoculated to the plants (Alkan et al. 2004; Jansa et al. 2008; Symanczik et al. 2015; Arruda et al. 2022). By contrast, in this study, we quantified native AMF from natural soil using the broad range primers AMG1F (Hewins et al. 2015) and AM1 (Helgason et al. 1998) which target the 18S rDNA sequence.

We found a strong correlation between the microscopic and qPCR quantification of AMF in tomato and the two wheat varieties Colmetta and Wiwa, but no correlation in leek (Fig. 1). The lack of correlation between microscopy and qPCR in leek has previously been reported (Gamper et al. 2008). In our pot experiment with leek, AMF abundance as measured with the qPCR method was not affected by P treatment (Fig. 2). By contrast, the total colonization as measured by microscopy was more uniform for leek than for the other two crops (Fig. 1), which may have contributed to the lack of correlation between the two methods. Additionally, we used root drying as a conservation method prior to DNA extraction. Recent research by Finn et al. (2023), however, has demonstrated that drying soil samples can have adverse effects on microbiome analysis when compared to various freezing approaches. It is worth noting that leek, with its distinct root morphology compared to wheat and tomatoes, may respond differently to drying, potentially influencing the composition of its associated microbiome and consequently affecting our qPCR results. Generally, freezing is the preferred method for sample conservation prior to microbiome analysis, although its feasibility may depend on the circumstances.

Several studies reported differences in the correlation between microscopy and qPCR depending on crop species (Alkan et al. 2004; Arruda et al. 2022). Alkan et al. (2004) found stronger correlation between microscopy and qPCR for tomato (family of *Solanaceae*) compared to *Medicago truncatula* (*Fabaceae*) using primers specific for *Glomus intraradices*. Arruda et al. (2022) also observed a stronger correlation for *Brachiaria* (*Poaceae*) than for *Crotalaria* (*Fabaceae*) using the AMF broad range primers FLR3 and FLR4 targeting the ribosomal large subunit (rLSU). Alkan et al. (2004) and Arruda et al. (2022) both suggested that differences in visibility of AMF structures inside the roots of different plants might explain the discrepancy observed, thus highlighting the disadvantage of the microscopic method. Moreover, the lack of correlation between microscopy and qPCR might be due to the differences in nuclei present in the different AMF structures (Pivato et al. 2007; Gamper et al. 2008); vesicles have been shown to contain a higher number of nuclei than hyphae or arbuscules (Bonfante-Fasolo et al. 1987; Gamper et al. 2008). Additionally, different AMF species are known to produce a high or low number of vesicles. Similarly, differences in nuclei concentrations are expected across AMF and along their developmental stages (Clapp et al. 2003; Sanders 2004). Accordingly, we speculate that the lack of correlation between microscopy and qPCR observed in leek results from (i) small difference in AMF root colonization found among P treatments and (ii) the AMF community composition colonizing the leek roots, which is expected to differ from that of tomato and wheat. One advantage of quantifying AMF root colonization with qPCR is the ability of using the PCR product for sequencing, thus reducing the number of techniques needed to assess the AMF-plant symbiosis. Regrettably, as the goal of this study was not to assess the AMF community composition, we did not sequence the PCR product.

We further evaluated the applicability of the spiking method developed by Thonar et al. (2012). This method involves the addition of a plasmid containing a DNA fragment of the cassava mosaic virus (internal standard, IS) before DNA extraction. This technique was developed to assess the DNA extraction efficiency and PCR amplification inhibition during AMF quantification. Our results revealed that spiking did not change the outcome of the qPCR method using the primer pair AMG1F and AM1. Furthermore, the spiking method duplicates the number of reactions needed for analyses. In addition, we argue that the extraction of DNA from fungal cells might not behave the same as the DNA extraction from a plasmid. Furthermore, qPCR cycling conditions for each primer pair are different; thus, amplification inhibition might not be the same. Moreover, if the PCR product also is used for sequencing, the spike sequences may alter the original community composition as shown by (Zhang et al. 2022). For this reason and to reduce the quantity of reagents needed, we advise to apply the spiking method only to a subset of samples to control for amplification inhibition during qPCR (Madueño et al. 2018).

Here, we used absolute qPCR instead of relative qPCR which was used in our previous paper (Bodenhausen et al. 2021). Absolute qPCR offers several advantages compared to relative qPCR including a reduction in the number of reactions required by half. In relative qPCR, the host genes must be quantified in a separate reaction, which requires additional time and labor. By reducing the number of reactions needed, throughput is increased, making it possible to analyze many samples in parallel. Furthermore, when studying plant communities or different crops and their interaction with AMF, finding a generic primer pair that amplifies DNA from all investigated plants with the same efficiency can be challenging. Although a primer has been designed to target the plant ITS region which was shown to exclude fungal reads (Cheng et al. 2016), to the best of our knowledge, it has not yet been tested for the analysis of the AMF-plant symbiosis. Therefore, we chose to use absolute qPCR in this study, which allowed us to quantify the absolute abundance of AMF without the need for plant-specific primers.

In this study, we employed the “magnified intersection” method to quantify root colonization (McGonigle et al. 1990). This approach has been widely used and allows for basic assessment of AMF colonization. It has some limitations, however, such as the lack of fine categorization of the different intensities of colonization. In contrast, the “Trouvelot” method offers a refined categorization of root colonization (Trouvelot et al. 1986). The latter method has shown improved agreement with results obtained by micrography and image analysis (Kokkoris et al. 2019). Furthermore, recent publications have highlighted the potential of artificial intelligence methods particularly deep learning approaches (Evangelisti et al. 2021) or convolutional neural networks (Muta et al. 2022) to revolutionize AMF quantification. These advanced techniques offer several advantages, including a reduction in time and decreased dependence on subjective observer interpretations. It is important for additional researchers to rigorously test these methods, however, to ensure their accuracy and applicability across a range of plant-fungal systems.

We conclude that the primer pair AMG1F and AM1 can be used for the quantification of AMF root colonization in wheat and tomato, but not leek. The failure of the method to effectively quantify colonization in leek leaves some open questions, such as the sensitivity of qPCR to observe only small changes in colonization and the potential impact of human error in the traditional microscopy approach. The qPCR method is time-efficient as we demonstrated in our previous paper, where we estimated that the qPCR method takes half the time of the microscopy method (Bodenhausen et al. 2021). A further advantage of qPCR is that it is little dependent on the observer compared to microscopy (McGonigle et al. 1990). Finally, qPCR facilitates the assessment of the community composition as the PCR product can be sequenced easily, as we showed previously (Bodenhausen et al. 2021). On the other hand, microscopy offers some advantages, such as providing information on the type of structures present and their spatial distribution in roots. Thus, we acknowledge that both qPCR and microscopy have their advantages and disadvantages, and we suggest that the choice between the two should be based on the specific needs of the study. In some cases, a combination of both methodologies may be necessary to fully address the research question at hand. Given the importance of the symbiotic relationship between AMF and plants in promoting plant growth and health, the development of high-throughput methods to assess AMF abundance in crops has the potential to greatly enhance our ability to utilize them, ultimately contributing to increased sustainability in food production.

### Supplementary Information

Below is the link to the electronic supplementary material.

Supplementary file1 (DOCX 42079 KB)

## References

[CR1] Abbott LK (1982). Comparative anatomy of vesicular-arbuscular mycorrhizas formed on subterranean clover. Aust J Bot.

[CR2] Alguacil MM, Torrecillas E, García-Orenes F, Roldán A (2014). Changes in the composition and diversity of AMF communities mediated by management practices in a Mediterranean soil are related with increases in soil biological activity. Soil Biol Biochem.

[CR3] Alkan N, Gadkar V, Coburn J (2004). Quantification of the arbuscular mycorrhizal fungus Glomus intraradices in host tissue using real-time polymerase chain reaction. New Phytol.

[CR4] Arruda B, Rodrigues YF, Herrera WFB et al (2022) Experimental validation under controlled conditions of real time PCR to quantify arbuscular mycorrhizal colonization in root. J Microbiol Methods 192. 10.1016/j.mimet.2021.106382 10.1016/j.mimet.2021.106382 34808146

[CR5] Badri A, Stefani FOP, Lachance G (2016). Molecular diagnostic toolkit for Rhizophagus irregularis isolate DAOM-197198 using quantitative PCR assay targeting the mitochondrial genome. Mycorrhiza.

[CR6] Boddington CL, Dodd JC (2000). The effect of agricultural practices on the development of indigenous arbuscular mycorrhizal fungi. I. Field studies in an Indonesian ultisol. Plant Soil.

[CR7] Bodenhausen N, Deslandes-Hérold G, Waelchli J (2021). Relative qPCR to quantify colonization of plant roots by arbuscular mycorrhizal fungi. Mycorrhiza.

[CR8] Bonfante-Fasolo P, Berta G, Fusconi A (1987). Distribution of nuclei in a VAM fungus during its symbiotic phase. Trans Br Mycol Soc.

[CR9] Brankatschk R, Bodenhausen N, Zeyer J, Burgmann H (2012). Simple absolute quantification method correcting for quantitative PCR efficiency variations for microbial community samples. Appl Environ Microbiol.

[CR10] Cheng T, Xu C, Lei L (2016). Barcoding the kingdom Plantae: new PCR primers for ITS regions of plants with improved universality and specificity. Mol Ecol Resour.

[CR11] Clapp JP, Helgason T, Daniell TJ (2003). Genetic Studies of the Structure and Diversity of Arbuscular Mycorrhizal Fungal Communities.

[CR12] Evangelisti E, Turner C, McDowell A (2021). Deep learning-based quantification of arbuscular mycorrhizal fungi in plant roots. New Phytol.

[CR13] Finn DR, Schroeder J, Samad MS et al (2023) Importance of sample pre-treatments for the DNA-based characterization of microbiomes in cropland and forest soils. Soil Biol Biochem 109077

[CR14] Gamborg OL, Wetter LR (1975) Plant tissue culture methods. National Research Council of Canada Prairie Regional Laboratory.

[CR15] Gamper HA, Young JPW, Jones DL, Hodge A (2008). Real-time PCR and microscopy: are the two methods measuring the same unit of arbuscular mycorrhizal fungal abundance?. Fungal Genet Biol.

[CR16] Giovannetti M, Mosse B (1980). An evaluation of techniques for measuring vesicular arbuscular mycorrhizal infection in roots. New Phytol.

[CR17] Gollotte A, Van Tuinen D, Atkinson D (2004). Diversity of arbuscular mycorrhizal fungi colonising roots of the grass species Agrostis capillaris and Lolium perenne in a field experiment. Mycorrhiza.

[CR18] Graham JH, Hodge NC, Morton JB (1995). Fatty acid methyl ester profiles for characterization of glomalean fungi and their endomycorrhizae. Appl Environ Microbiol.

[CR19] Green HC, Field KG (2012). Sensitive detection of sample interference in environmental qPCR. Water Res.

[CR20] Helgason T, Daniell TJ, Husband R (1998). Ploughing up the wood-wide web?. Nature.

[CR21] Heller WP, Carrara JE (2022). Multiplex qPCR assays to distinguish individual species of arbuscular mycorrhizal fungi from roots and soil. Mycorrhiza.

[CR22] Hewins CR, Carrino-Kyker SR, Burke DJ (2015). Seasonal variation in mycorrhizal fungi colonizing roots of Allium tricoccum (wild leek) in a mature mixed hardwood forest. Mycorrhiza.

[CR23] Jansa J, Smith FA, Smith SE (2008). Are there benefits of simultaneous root colonization by different arbuscular mycorrhizal fungi?. New Phytol.

[CR24] Kahiluoto H, Ketoja E, Vestberg M, Saarela I (2001). Promotion of AM utilization through reduced P fertilization 2. Field Studies Plant Soil.

[CR25] Keymer A, Gutjahr C (2018). Cross-kingdom lipid transfer in arbuscular mycorrhiza symbiosis and beyond. Curr Opin Plant Biol.

[CR26] Kokkoris V, Pogiatzis A, Hart MM (2019). Contrasting common measures of arbuscular mycorrhizal fungal root colonization. J Microbiol Methods.

[CR27] Lee J, Lee S, Young JPW (2008). Improved PCR primers for the detection and identification of arbuscular mycorrhizal fungi. FEMS Microbiol Ecol.

[CR28] Madueño L, Paul C, Junier T (2018). A historical legacy of antibiotic utilization on bacterial seed banks in sediments. PeerJ.

[CR29] McGonigle TP, Miller MH, Evans DG (1990). A new method which gives an objective measure of colonization of roots by vesicular-arbuscular mycorrhizal fungi. New Phytol.

[CR30] Merryweather J, Fitter A (1998). The arbuscular mycorrhizal fungi of Hyacinthoides non-scripta I. Diversity of Fungal Taxa New Phytol.

[CR31] Murphy J, Riley JP (1958). A single-solution method for the determination of soluble phosphate in sea water. J Mar Biol Assoc UK.

[CR32] Muta K, Takata S, Utsumi Y (2022). TAIM: tool for analyzing root images to calculate the infection rate of arbuscular mycorrhizal fungi. Front Plant Sci.

[CR33] Nagy R, Drissner D, Amrhein N (2009). Mycorrhizal phosphate uptake pathway in tomato is phosphorus-repressible and transcriptionally regulated. New Phytol.

[CR34] Olsson PA, Bååth E, Jakobsen I, Söderström B (1995). The use of phospholipid and neutral lipid fatty acids to estimate biomass of arbuscular mycorrhizal fungi in soil. Mycol Res.

[CR35] Olsson PA, Francis R, Read D, Söderström B (1998). Growth of arbuscular mycorrhizal mycelium in calcareous dune sand and its interaction with other soil microorganisms as estimated by measurement of specific fatty acids. Plant and Soil.

[CR36] Parniske M (2008). Arbuscular mycorrhiza: the mother of plant root endosymbioses. Nat Rev Microbiol.

[CR37] Pfaffl MW (2001). A new mathematical model for relative quantification in real-time RT-PCR. Nucleic Acids Res.

[CR38] Phillips JM, Hayman DS (1970). Improved procedures for clearing roots and staining parasitic and vesicular-arbuscular mycorrhizal fungi for rapid assessment of infection. Trans Br Mycol Soc.

[CR39] Pivato B, Mazurier S, Lemanceau P (2007). Medicago species affect the community composition of arbuscular mycorrhizal fungi associated with roots. New Phytol.

[CR40] R Core Team (2020) R: a language and environment for statistical computing

[CR41] Sanders IR (2004). Plant and arbuscular mycorrhizal fungal diversity – are we looking at the relevant levels of diversity and are we using the right techniques?. New Phytol.

[CR42] Schmittgen TD, Livak KJ (2008). Analyzing real-time PCR data by the comparative CT method. Nat Protoc.

[CR43] Smith SE, Read DJ (2010). Mycorrhizal Symbiosis.

[CR44] Symanczik S, Courty P-E, Boller T (2015). Impact of water regimes on an experimental community of four desert arbuscular mycorrhizal fungal (AMF) species, as affected by the introduction of a non-native AMF species. Mycorrhiza.

[CR45] Thonar C, Erb A, Jansa J (2012). Real-time PCR to quantify composition of arbuscular mycorrhizal fungal communities-marker design, verification, calibration and field validation. Mol Ecol Resour.

[CR46] Trouvelot A, Kough J, Gianinazzi-Pearson V (1986) Mesure du taux de mycorhization VA d’un système radiculaire. Recherche de méthodes d’estimation ayant une signification fonctionnelle. Physiological and genetical aspects of mycorrhizae: Proceedings of the 1st european symposium on mycorrhizae 1:217–221

[CR47] van der Heijden MG, Martin FM, Selosse MA, Sanders IR (2015). Tansley review Mycorrhizal ecology and evolution : the past, the present, and the future. New Phytol.

[CR48] Vierheilig H, Schweiger P, Brundrett M (2005). An overview of methods for the detection and observation of arbuscular mycorrhizal fungi in roots. Physiol Plant.

[CR49] Voříšková A, Jansa J, Püschel D (2017). Real-time PCR quantification of arbuscular mycorrhizal fungi: does the use of nuclear or mitochondrial markers make a difference?. Mycorrhiza.

[CR50] Walder F, Van Der Heijden MGA (2015). Regulation of resource exchange in the arbuscular mycorrhizal symbiosis. Nat Plants.

[CR51] Wickham H (2016) ggplot 2: elegant graphics for data analysis

[CR52] Zhang M, Zhang L, Huang S et al (2022) Assessment of spike-AMP and qPCR-AMP in soil microbiota quantitative research. Soil Biol Biochem 166:108570. 10.1016/j.soilbio.2022.108570

