# Supplementary material for: Quantification of arbuscular mycorrhizal fungi root colonization in wheat, tomato, and leek using absolute qPCR
Source: Mycorrhiza. 2023 Aug 30;33(5-6):387–97. doi: 10.1007/s00572-023-01122-8 (PMC10752845; doi:10.1007/s00572-023-01122-8)
Supplement: Supplementary file 1 — Supplementary file1 (DOCX 42079 KB) [file 572_2023_1122_MOESM1_ESM.docx]

# Supplementary material

## Title of the manuscript

Quantification of arbuscular mycorrhizal fungi root colonization in wheat, tomato and leek using absolute qPCR

## Authors

Andrea Corona Ramírez, Sarah Symanczik, Tabea Gallusser, Natacha Bodenhausen

This file includes:

Figures S1 to S6

Tables S1 to S4

## Figures

**
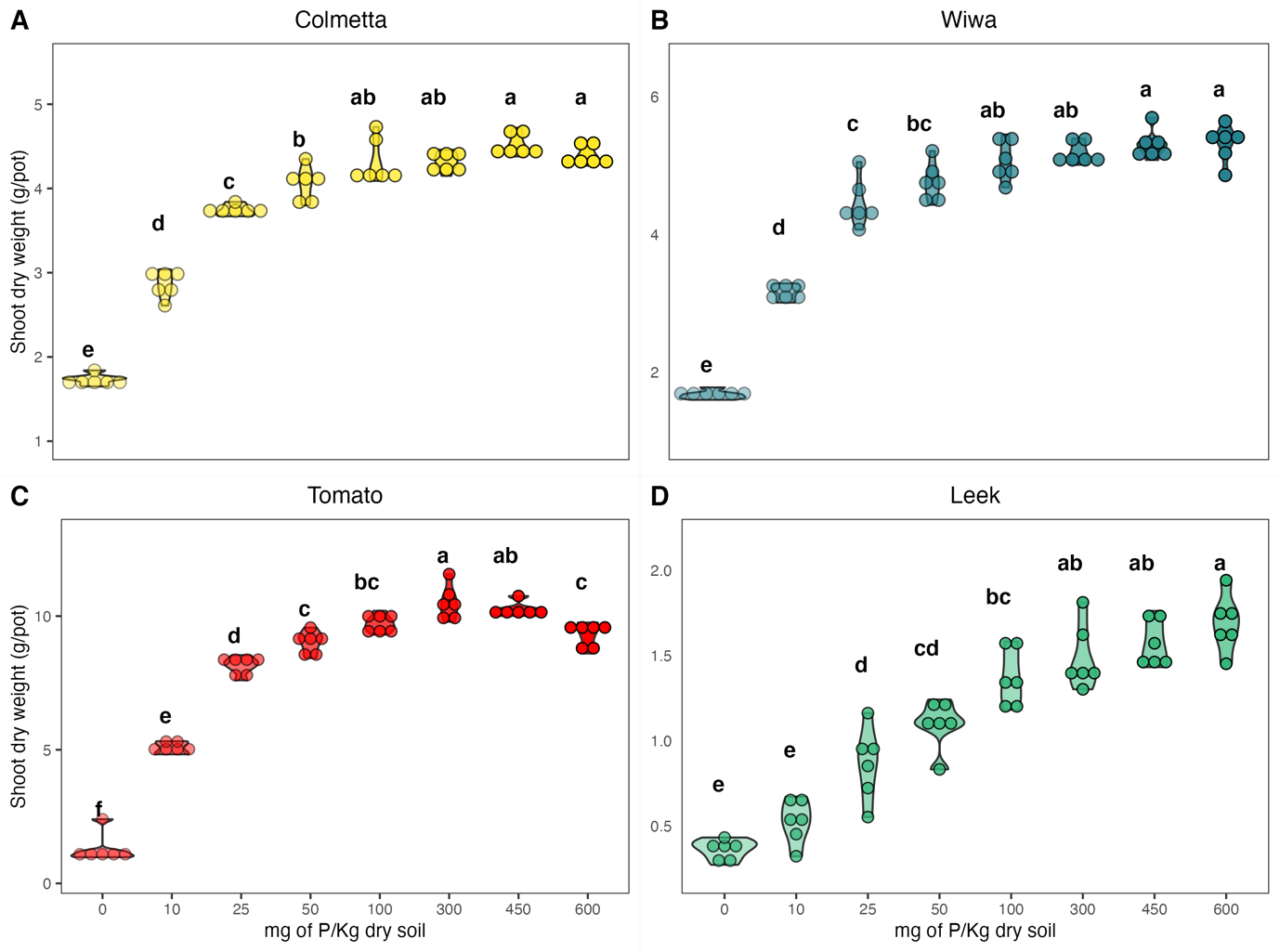
**

**Fig. S1.** Effect of the phosphorus (P) fertilization gradient on the shoot dry weight of the winter wheat varieties Colmetta (A) and Wiwa (B), tomato (C) and leek (D)**.** Each point represents a replicate, and the colour intensity increases with increasing P fertilization level. Treatments topped by the same letter do not differ significantly by Tukey’s honestly significant difference test.

**
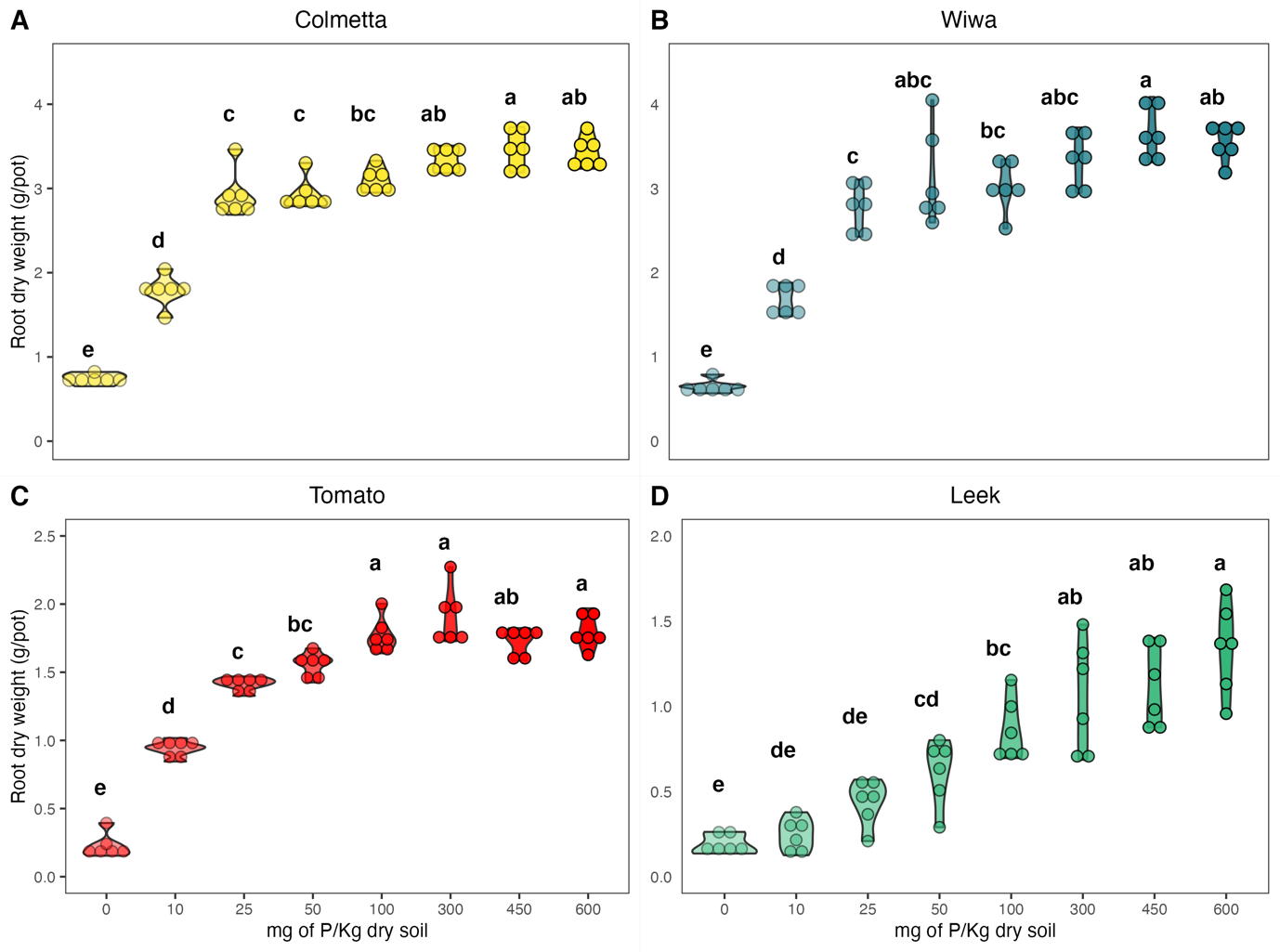
**

**Fig. S2.** Effect of the phosphorus (P) fertilization gradient on the root dry weight of the winter wheat varieties Colmetta (A) and Wiwa (B), tomato (C) and leek (D)**.** Each point represents a replicate, and the colour intensity increases with increasing P fertilization level Treatments topped by the same letter do not differ significantly by Tukey’s honestly significant difference test.

**
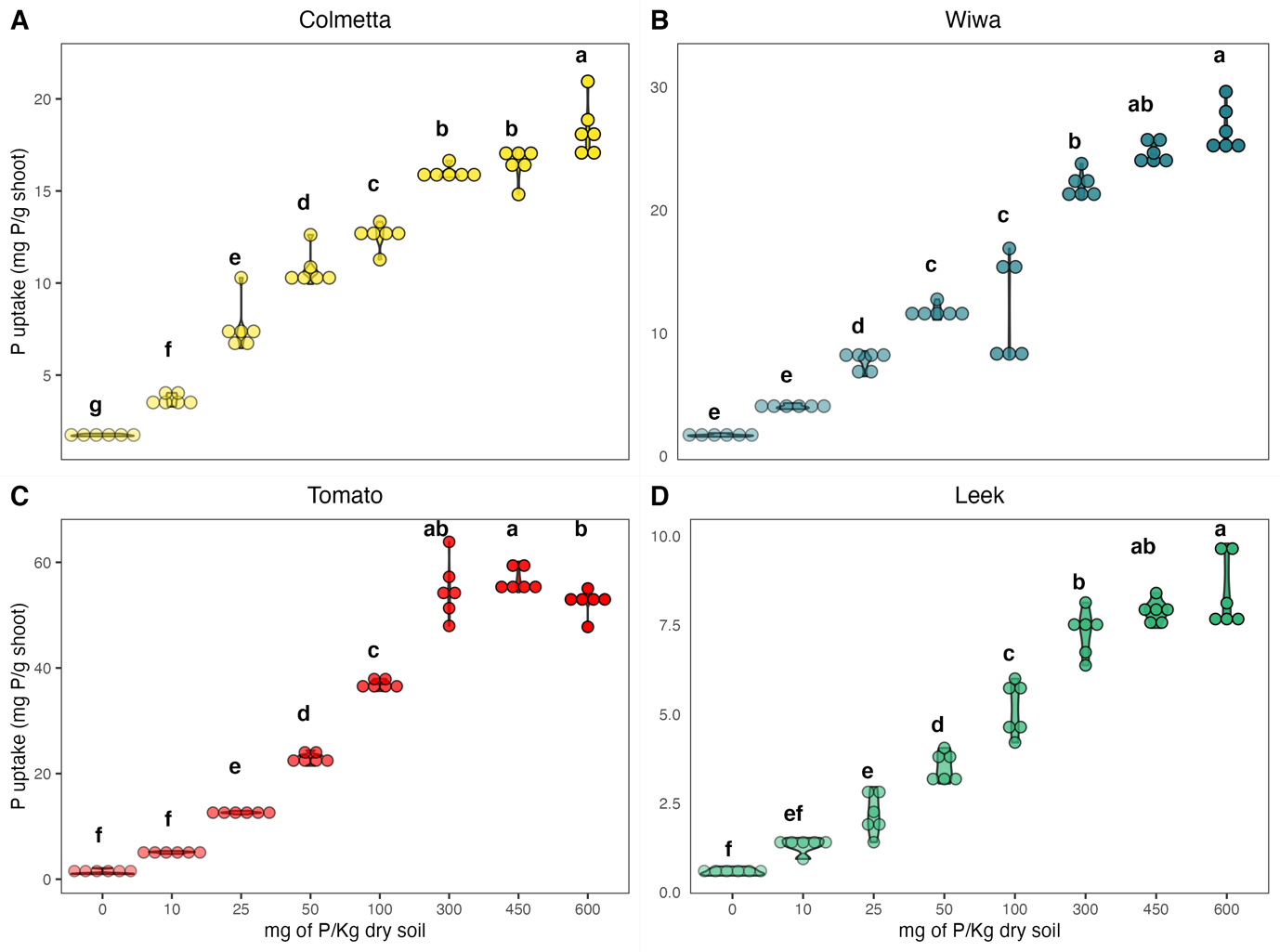
**

**Fig. S3.** Phosphor (P) plant uptake of the winter wheat varieties Colmetta (A) and Wiwa (B), tomato (C) and leek (D). Each point represents a replicate, and the colour intensity increases with increasing P fertilization level. Treatments topped by the same letter do not differ significantly by Tukey’s honestly significant difference test.


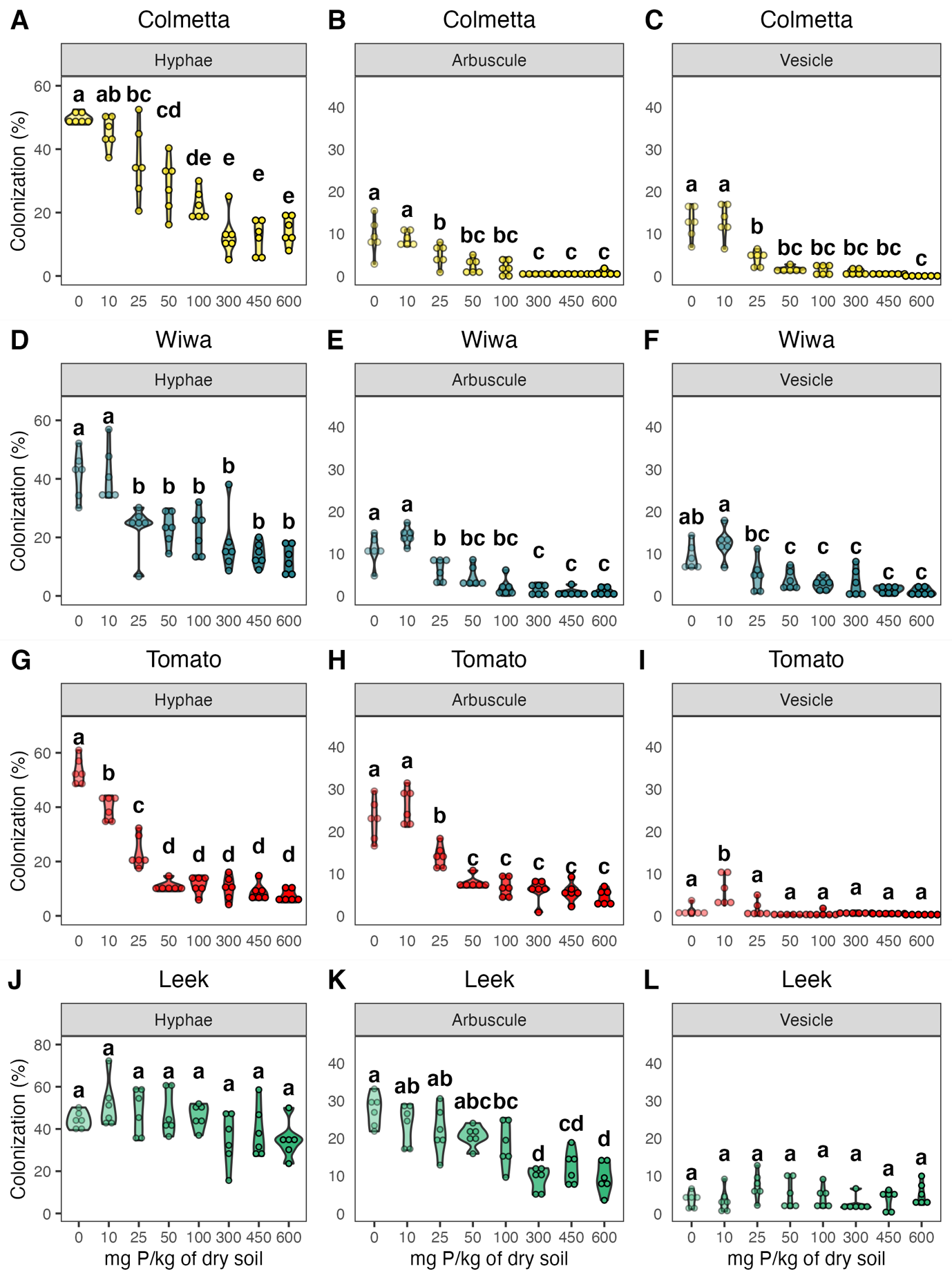
**Fig. S4.** Composition of the AMF root colonization the winter wheat varieties Colmetta (A) and Wiwa (B), tomato (C) and leek (D). Three plots per crop are presented, each one showing the abundance of hyphae, arbuscule or vesicle in plant roots. Each point represents a replicate, and the colour intensity increases with increasing P fertilization level. Treatments topped by the same letter do not differ significantly by Tukey’s honestly significant difference test.

**
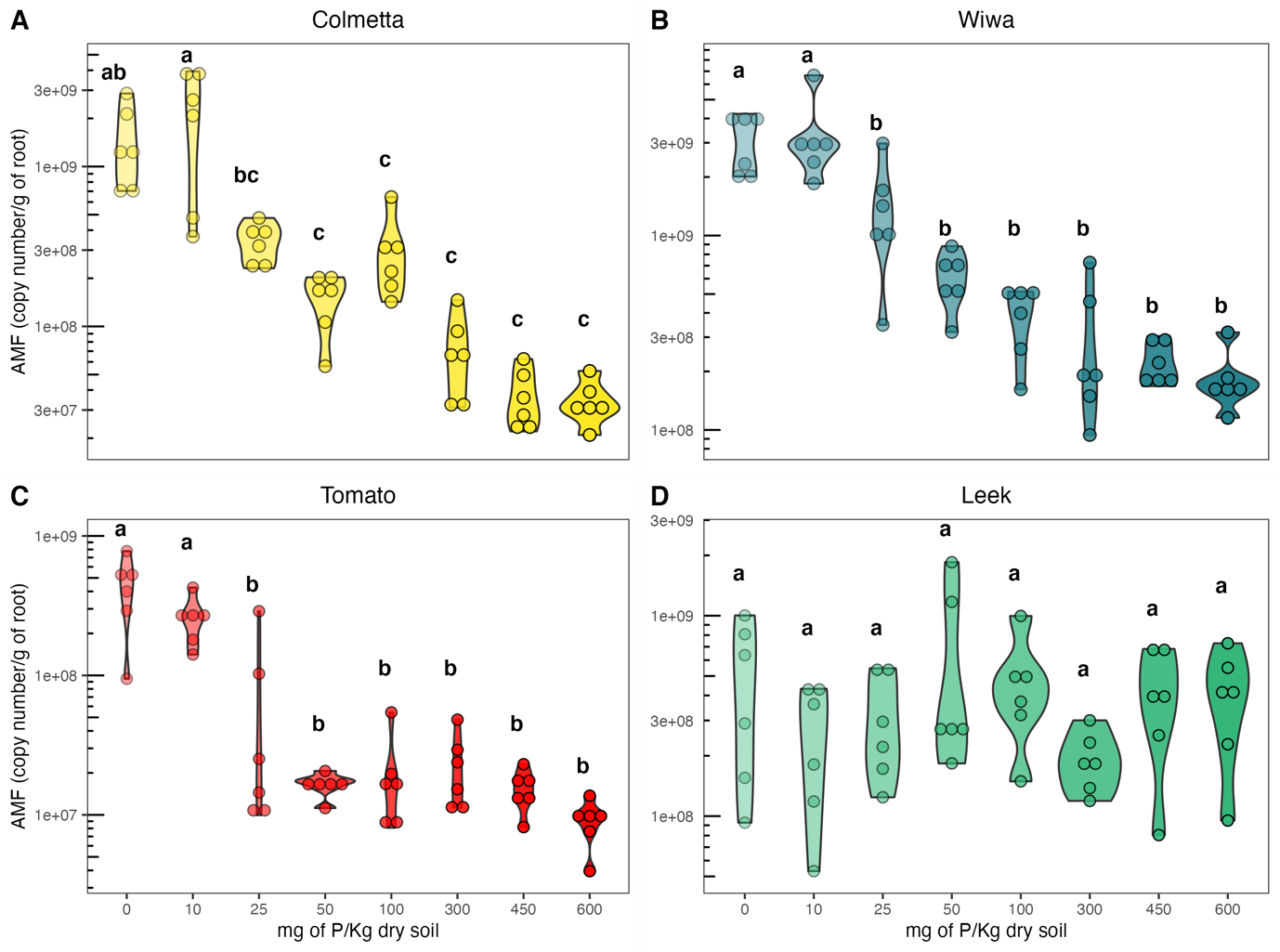
**

**Fig. S5.** Arbuscular mycorrhizal fungal (AMF) copy numbers in roots of the winter wheat varieties Colmetta (A) and Wiwa (B), tomato (C), and leek (D) assessed by quantitative polymerase chain reaction (qPCR), corrected with the spiking method. Each point represents a replicate, and the colour intensity increases with increasing P fertilization level. Treatments topped by the same letter do not differ significantly by Tukey’s honestly significant difference test.


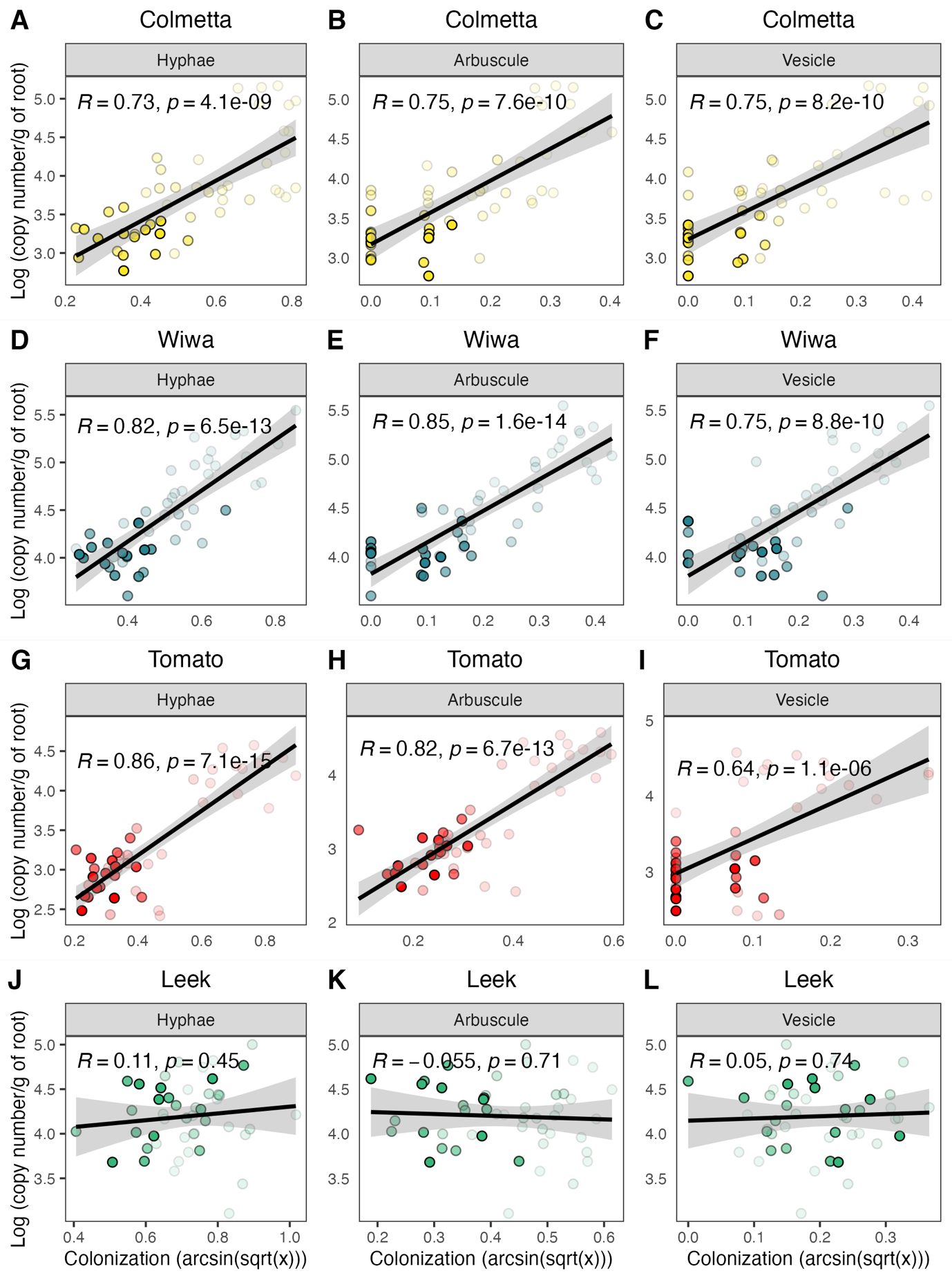
**Fig. S6.** Correlation between the different AMF structures (y-axis) and the abundance of AMF copy numbers quantified by qPCR (x-axis) in the winter wheat varieties Colmetta (A, B, C), Wiwa (D,E,F), tomato (G, H, I) and leek (J, K, L). Each point represents a replicate, and the color intensity increases with increasing phosphorus fertilization level. R is the Pearson correlation coefficient with associated P-value.

## Tables

**Table S1.** Soil properties. Abbreviations: CAT stands for Extraction in calcium chloride-diethylenetriaminepentaacetic acid (DTPA) solution (1:9, w:v), KW stands for extraction in aqua regia, ICP-OES stands for inductively coupled plasma optical emission spectroscopy, DL stands for double lactate extraction.

| Dompierre 2015 | | |
| --- | --- | --- |
| Date | 13.12.16 | |
| Parameter | Units | Value |
| pH in H_2_O |  | 7.7 |
| Humus, Elemental analysis | % | 2.18 |
| Nitrogen (N), Elemental analysis | % | 0.179 |
| Sulfur (S), Elemental analysis | % | 0.021 |
| Sand (63 - 2000 µm) | % | 43.5 |
| Silt (2 - 63 µm) | % | 41.7 |
| Clay (< 2 µm) | % | 14.8 |
| Carbonates | % | 4.57 |
| Iron (Fe), CAT-Extract | mg/kg | 22.0 |
| Copper (Cu), CAT-Extract | mg/kg | 2.03 |
| Magnesium (Mg), CAT-Extract | mg/kg | 59 |
| Manganese (Mn), CAT-Extract | mg/kg | 20.5 |
| Zinc (Zn), CAT-Extract | mg/kg | 0.85 |
| Total Carbon (C) Elemental analysis | % | 3.01 |
| Phosphor (P), Olsen | mg/kg | 10.3 |
| Potassium (K), CAT-Extract | mg/kg | 30 |
| Molybdenum (Mo), CAT-Extract | mg/kg | <0,010 |
| P2O5 (DL-Extract) | mg/100g |  |
| Boron (B), ICP-OES KW | mg/kg | 14.3 |
| Calcium (Ca), ICP-OES KW | mg/kg | 56,844 |
| Iron (Fe), ICP-OES KW | mg/kg | 18,478 |
| Potassium (K), ICP-OES KW | mg/kg | 2,237 |
| Copper (Cu), ICP-OES KW | mg/kg | 18.6 |
| Magnesium (Mg), ICP-OES KW | mg/kg | 8,427 |
| Manganese (Mn), ICP-OES KW | mg/kg | 583 |
| Phosphor (P), ICP-OES KW | mg/kg | 623 |
| Zinc (Zn), ICP-OES KW | mg/kg | 46.2 |

**Table S2**. Cloned PCR fragment of Rhizoglomus irregulare (18S region; AML1/2 primer pair)

| Forward primer | Reverse primer | Sequence (rev) |
| --- | --- | --- |
| AML1  (Lee et al. 2008) | AML2  (Lee et al. 2008) | ATCAACTTTCCGATGGTAGGATAGAGGCCTACCATGGTGGTAACGGGTAACGGGGTGTTAGGGCACGACACCGGAGAGGGAGCCTGAGAAACGGCTACCACATCCAAGGATGGCAGCAGGCGCGCAAATTACCCAATCCCGACACGGGGAGGTAGTGACAATAAATAACAATACGGGGTTCTTTCGGATCTCGTAATTGGAATGAGTACAATTTAAATCTCTTAACGAGGAACAATTGGAGGGCAAGTCTGGTGCCAGCAGCCGCGGTAATTCCAGCTCCAATAGCGTATATTAAAGTTGTTGCAGTTAAAAAGCTTGTAGTTAAATTTCGGGGTTAGTAGGTTGGTCATGCCTCCGGTATGTACTGATCTCACTGATTCCTCCTTCCTTATGAACCGTAATGCCATTAATTTGGTGTTGCGGGGAATTTGGACTGTTACTTTGAAAAAATTAGAGTGTTTAAAGCAAGCTAACGCTTGAATACATTAGCATGGAATAATGAAATAGGACGTTCGATCCTATTTTGTTGGTTTCTAGGATTGACGTAATGATTAATAGGGATAGTTGGGGGCATTAGTATTCAATTGTCAGAGGTGAAATTCTTGGATTTATTGAAGACTAACTACTGCGAAAGCATTTGCCAAGGATGTTTTCATTAATCAAGAACGAAAGTTAGGGGATCGAAGACGATCAGATACCGTCGTAGTCTTAACCATAAACTATGCCGACTAGGGATCGGATGATGTTAATTTTTTAATGACTCATTCGGCGCCTTACGGGAAACCAAAGTGTTTGGGTTC |

**Table S3.** Effect of phosphorus fertilization on shoot dry weight, colonization of arbuscular mycorrhizal fungi (AMF) assessed via microscopy and quantitative polymerase chain reaction (qPCR) for the winter wheat varieties Colmetta and Wiwa, tomato and leek. F and p values were obtained from one-way analysis of variance (ANOVA).

| Factor | Colmetta | | Wiwa | | Tomato | | Leek | |
| --- | --- | --- | --- | --- | --- | --- | --- | --- |
|  | F value | p-value | F value | p-value | F value | p-value | F value | p-value |
| Shoot dry weight | 245.22 | <0.001 | 186.371 | <0.001 | 344.821 | <0.001 | 60.049 | <0.001 |
| Microscopy | 52.351 | <0.001 | 22.942 | <0.001 | 75.67 | <0.001 | 8.99 | <0.001 |
| qPCR | 15.431 | <0.001 | 22.942 | <0.001 | 19.212 | <0.001 | 1.179 | 0.336 |

**Table S4.** Effect of phosphorus fertilization on the abundance of arbuscular mycorrhizal fungal (AMF) structures quantified in roots of the winter wheat varieties Colmetta and Wiwa, tomato and leek. F and p value were obtained from one-way analysis of variance (ANOVA).

| AMF structure | Colmetta | | Wiwa | | Tomato | | Leek | |
| --- | --- | --- | --- | --- | --- | --- | --- | --- |
|  | F value | p-value | F value | p-value | F value | p-value | F value | p-value |
| Hyphae | 29.662 | <0.001 | 13.002 | <0.001 | 110.502 | <0.001 | 2.479 | <0.05 |
| Arbuscule | 18.864 | <0.001 | 32.499 | <0.001 | 47.768 | <0.001 | 12.734 | <0.001 |
| Vesicle | 38.254 | <0.001 | 13.488 | <0.001 | 11.127 | <0.001 | 1.494 | 0.197 |
